# Current Status and Future Outlook of Additive Manufacturing Technologies for the Reconstruction of the Trachea

**DOI:** 10.3390/jfb14040196

**Published:** 2023-04-02

**Authors:** Hwa-Yong Lee, Jin Woo Lee

**Affiliations:** 1 Division of Science Education, Kangwon National University, Chuncheon 24341, Republic of Korea; 2 Department of Molecular Medicine, College of Medicine, Gachon University, Incheon 21999, Republic of Korea

**Keywords:** trachea, reconstruction, 3D printing, bioprinting, scaffold

## Abstract

Tracheal stenosis and defects occur congenitally and in patients who have undergone tracheal intubation and tracheostomy due to long-term intensive care. Such issues may also be observed during tracheal removal during malignant head and neck tumor resection. However, to date, no treatment method has been identified that can simultaneously restore the appearance of the tracheal skeleton while maintaining respiratory function in patients with tracheal defects. Therefore, there is an urgent need to develop a method that can maintain tracheal function while simultaneously reconstructing the skeletal structure of the trachea. Under such circumstances, the advent of additive manufacturing technology that can create customized structures using patient medical image data provides new possibilities for tracheal reconstruction surgery. In this study, the three-dimensional (3D) printing and bioprinting technologies used in tracheal reconstruction are summarized, and various research results related to the reconstruction of mucous membranes, cartilage, blood vessels, and muscle tissue, which are tissues required for tracheal reconstruction, are classified. The prospects for 3D-printed tracheas in clinical studies are also described. This review serves as a guide for the development of artificial tracheas and clinical trials using 3D printing and bioprinting.

## 1. Introduction

The trachea is a functional skeleton composed of two tissues: the exoskeleton connected by a cartilage ring and the inner respiratory mucosal epithelium. Tracheal stenosis and defects occur congenitally at a rate of 1 in 64,500 newborns. Furthermore, they may be observed in patients who have undergone tracheal intubation and tracheostomy due to long-term intensive care and tracheal removal during malignant head and neck tumor resection [1]. In the case of anastomosis, in which a damaged part of the trachea is removed, the remaining normal trachea is connected; if more than 50% of the trachea in an adult and 30% of the trachea in a child shows tracheal defects, surgery is impossible [2,3,4]. However, to date, no treatment methods are available that can simultaneously restore the appearance of the tracheal skeleton while maintaining respiratory function in patients with tracheal defects. Therefore, a method that can maintain tracheal function while simultaneously reconstructing the skeletal structure of the trachea is urgently required.

Until now, various methods such as decellularized tracheal constructs, electrospun fibrous constructs, and mesh tubes with biomaterial sponges have been attempted for tracheal reconstruction [5,6,7,8,9]. In addition, attempts using stem cells and rigid scaffolds have also been conducted recently. However, in certain cases, the trachea is blocked due to respiratory mucosal epithelium insufficiency of the tracheal lumen, and survival of the patients who underwent surgery is not possible [10]. Under these circumstances, the advent of three-dimensional (3D) printing technology that can create customized structures using medical imaging data of patients, also known as ‘additive manufacturing,’ provides new possibilities in tracheal reconstruction surgery. In addition, 3D bioprinting technology that can print cells and the extracellular matrix (ECM) together is expected to show merit in improving tracheal reconstruction.

Various studies have been conducted since Zopf et al. reported the clinical application of a 3D-printed external airway splint in an eight-week-old infant with bronchomalacia in 2013 [11]. They used PCL as the 3D printing material. Unlike PLA and PLGA, PCL does not produce acid as a degradation product and has a relatively long degradation time. In 2015 and 2017, polycaprolactone (PCL)- and polyetherketoneketone (PEKK)-based external airway splints were fabricated and used for surgery, respectively [12,13]. In the surgical and follow-up results of 15 pediatric patients in 2019, most showed improved breathing [14].

**Figure 1:**
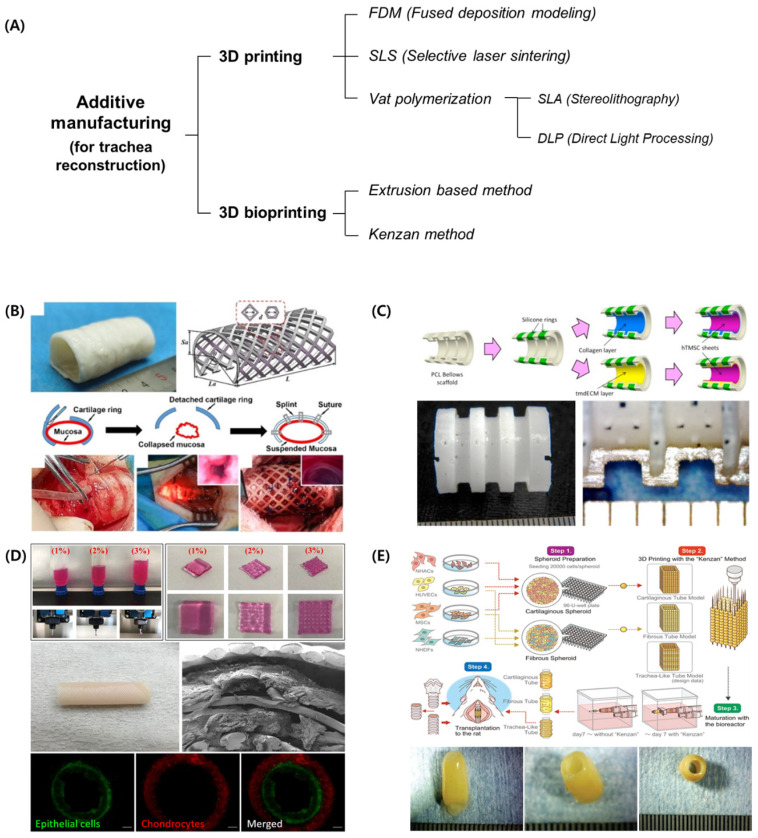
Additive manufacturing technologies utilized to reconstruct the trachea. (**A**) Classification of the additive manufacturing technologies. (**B**) Tracheal constructs using selective laser sintering. (**C**) Tracheal constructs using stereolithography. (**D**) Tracheal constructs using extrusion-based bioprinting. (**E**) Tracheal constructs using the Kenzan method. (Figures were reprinted with permission from Ref. [20], Elsevier [22,23]).

In the case of bioprinting, no clinical applications have been reported to date, but various studies conducted using medium- and large-sized animals have been reported. In 2017, a 3D-printed PCL graft with a partial ring was transplanted into pigs to mimic the tracheal cartilage with an anterior defect of 4 cm [15]. In 2019, a PCL-based 3D-printed columnar tracheal graft was fabricated, seeded with ear chondrocytes, and transplanted into goats [16]. In addition, experiments in rats and rabbits using bioprinting systems have been reported [17,18,19,20,21]. However, a complete 3D-bioprinted trachea that can be transplanted has not been reported. In this review, the 3D printing and bioprinting technologies utilized for tracheal reconstruction are summarized (Figure 1), and the current advances in research and the future outlook for the in vitro/in vivo printing of tracheas are outlined. This review serves as a guide for the development of artificial tracheas and clinical trials using 3D printing and bioprinting.

## 2. 3D-Printing-Based Technologies for Tracheal Reconstruction

### 2.1. 3D Printing

Unlike the existing production methods of cutting or removing materials, 3D printing, also called additive manufacturing technology, which builds 3D products by stacking many thin layers individually, has been studied since the 1980s [24,25,26]. Usually, developed 3D computer-aided design (CAD) data are cut for each layer, and two-dimensional sliced data are obtained subsequently. Then, using these sliced data, thin layered sheets are created for the formation of each layer and sequentially stacked to obtain the same 3D shape as that provided by the CAD data [27]. In addition, the manufacturing process is performed automatically, which is a characteristic feature of 3D printing. Overall, 3D printing technology can be divided into extrusion, photopolymerization, sintering, drawing, dropping, and lamination stages according to the accumulation method [28]. In addition, 3D printing can utilize most materials, including polymers, metals, paper, wood, food materials, and biomaterials.

**Figure 2:**
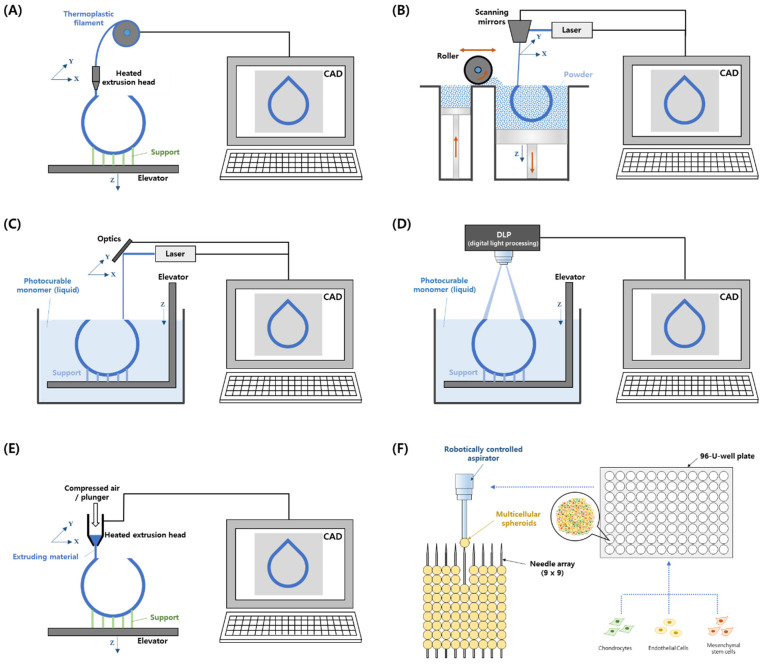
Schematic of additive manufacturing technologies for tracheal reconstruction. (**A**) Fused deposition modeling. (**B**) Selective laser sintering. (**C**) Stereolithography. (**D**) Direct light processing. (**E**) Extrusion-based bioprinting. (**F**) Kenzan method. CAD, Computer-aided design.

Recently, 3D printing technology combined with medical data, such as computed tomography and magnetic resonance imaging data, has innovated patient-specific medical devices and tissue-regeneration research [29,30]. In the early 2000s, a 3D printer was used to fabricate scaffolds as extracellular matrices [31,32,33,34,35], and a 3D bioprinter was used to regenerate target tissues by extruding cells onto the scaffold geometry [36,37,38,39]. In particular, for the reconstruction of the trachea, fused deposition modeling (FDM), selective laser sintering (SLS), and vat polymerization 3D printing technologies have been used (Figure 2). In addition, electrospinning has been combined with 3D printing technologies to enhance cell adhesion [40,41].

#### 2.1.1. FDM

FDM is a 3D printing method in which filament wires are extruded by feeders, or melted polymer granules are extruded by cylinders, screws, or pneumatics to fabricate complex 3D shapes. Therefore, FDM can utilize many thermoplastic materials developed by material science and industry [42]. The printing process of FDM is as follows: The thermoplastic material is melted by a heated extrusion nozzle, and the molten material is extruded onto a bed. The computer-controlled nozzle or bed moves in the X, Y, and Z directions, and a 3D scaffold is then built [43]. Similar to other 3D printing technologies, the properties of the scaffold can be modulated by controlling the printed line size, pore size, shape and interconnectivity, and scaffold design [44]. Because of the simple system configuration of the heating module and three-axis motion actuators, the FDM system is inexpensive and easy to maintain compared to other 3D printing systems. However, the resolution of FDM products and printing quality are relatively low.

#### 2.1.2. SLS

The powder bed fusion method is a 3D printing technology that builds a 3D structure by selectively fusing powder on the bed using thermal energy. Among these methods, SLS involves fabricating a 3D structure by sintering only specific parts of the powder using a high-power laser [45]. The SLS process can be divided into two main steps: powder flattening on the building bed using a roller or flattener and the sintering process of the designed layer by mediated by laser movements. The 3D scaffold is generated by repeating these two steps [46]. SLS uses polymeric, ceramic, and metal powders as materials for scaffold fabrication [47,48]. However, because this process uses thermal energy at high temperatures, it is difficult to use it with cells and biomolecules. In addition, the resolution of the scaffold is not high because of the limitation of powder size [49].

#### 2.1.3. Vat Photopolymerization–Stereolithography (SLA) and Direct Light Processing (DLP)

Vat photopolymerization is a type of 3D printing technique that uses a liquid photopolymer, and SLA and DLP are representative methods. In SLA, a laser is irradiated to a point on the surface of a liquid photopolymer layer, which is then cured by photopolymerization. Then, the designed layer is selectively solidified by the movement of the laser irradiation position. Subsequently, a 3D scaffold is manufactured using a layer-by-layer process [50]. In the case of DLP, planar light from a light source is selectively reflected, forming an imaged light layer [35,51,52]. By irradiating the light layer on the surface of a liquid polymer layer, the surface of the liquid photopolymer is cured simultaneously. Thereafter, the same process is performed to fabricate a 3D scaffold layer by layer. Therefore, DLP has an advantage in that its production speed is faster than that of SLA. Because vat photopolymerization can produce high-resolution and complex structures, it can demonstrate dimensional accuracy at both nano- and micro-scales. However, vat photopolymerization can use only photocrosslinkable materials, and post-curing is often used to improve mechanical strength [53,54].

#### 2.1.4. Electrospinning

Electrospinning is a method of producing fine fibers from a polymer solution using electrostatic force [55]. Electrospinning can be used to manufacture scaffolds by collecting electrospun fibers of nanometer to micrometer thickness. To produce electrospun fibers, a high DC voltage of several tens of kilovolts is required, and the solvent that dissolves the polymer must be easily evaporated during the electrospinning process. When the polymer solution, which is connected to the high-voltage source, is discharged through the capillary nozzle via an external force, it moves at a constant flow rate toward the ground/opposite polarity connected collector. Finally, continuous fibers are collected and stacked on a collector [56]. By controlling the applied voltage, solvent, polymer concentration, and emission amount, fibers of various sizes can be manufactured. This technology has the advantage of creating fibers using various polymers at room temperature. In addition, because electrospinning can achieve a very high surface-to-volume ratio, it can provide an environment for attaching cells.

### 2.2. 3D Bioprinting

3D Bioprinting is a technology that fabricates 3D structures via a layer-by-layer additive process of bioinks composed of biomaterials and/or cells [57,58]. As bioprinting uses cell components, a cell-culture process is needed along with the 3D printing process. In addition, air filters and temperature control systems are required to prevent contamination and increase the cell survival rate, respectively. To date, extrusion-based 3D bioprinting and the Kenzan method have been used to reconstruct tracheas [59,60,61].

#### 2.2.1. Extrusion-Based Bioprinting

Because extrusion-based printing comprises a three-axis stage and an extrusion module, it is easier to maintain than other bioprinting systems. Therefore, it is the most widely used method of bioprinting [62]. After loading the bioink into the syringe system, ink extrusion using pneumatic or mechanical (plunger or rotating screw) methods progresses at a predetermined location to form a single layer. A 3D structure is fabricated via an additive layer-by-layer process. In extrusion-based printing, a system is configured to enable the printing of thermoplastic polymers by mounting an FDM-type syringe along with a hydrogel, and 3D printing using multiple materials is rendered possible [57,63,64]. However, extrusion-based printing has the disadvantage of limiting the use of low-viscosity inks and nozzle-clogging issues owing to cell aggregation [65].

#### 2.2.2. Kenzan Method

The Kenzan method involves the creation of scaffold-free 3D cellular structures by inserting spheroids into fine-needle arrays according to the submitted data using a computer-controlled bioprinting system [19]. After preparing multicellular spheroids using cell culture plates, the spheroids are aspirated using a robotically controlled nozzle and placed into a needle array under computer control. After the spheroid insertion process is completed, 3D cellular structures mature inside a media-perfused bioreactor. 

## 3. Tracheal Reconstruction Using 3D Printing

### 3.1. External Splint

Since Zopf et al. implanted a 3D-printed airway splint to cure tracheobronchomalacia (TBM) in infants, studies on various types of external splints for humans and animals have been conducted. In 2013, Zopf et al. first fabricated a patient-specific implant based on SLS 3D printing technology, bioresorbable polycaprolactone, and high-resolution images, and no problems related to the tracheal splint were observed for one year after implantation [11]. Morrison et al. implanted 3D-printed external tracheal splints in three infants with severe TBM. Patient-specific splints were fabricated using PCL [12]. The splinted tracheas showed improved patency and continued growth of the primary airway. Follow-up after implantation for up to 38 months revealed no problems related to the implanted splint. They also proposed a splint with a high Young’s modulus and tensile strength using PEKK [13] for patients with adult-phenotype TBM. The wall thickness of the splint was reduced to make the device lighter, and the opening angle of the splint was increased to facilitate placement. The tracheal hydraulic diameter increased by 3.1 mm one month after implantation. Huang et al. fabricated an implanted PCL scaffold using 3D printing of FDM in a female patient with tracheomalacia [66]. After implantation, the inner diameter of the cavity was increased from 0.3 to 1.0 cm, and the cross-sectional area was increased by 4–5 times. During the first three months of follow-up, the patient showed improvement in breathing and physical strength. In addition, Les et al. reported the clinical safety and efficacy of the implantation of a 3D-printed tracheal splint in 15 patients from 2012 to 2018 [14]. Among these patients, 11 returned home, 3 died, and 1 who underwent surgery in 2018 remained in the hospital.

Meanwhile, Zopf et al. tested the effectiveness of 3D-printed bioresorbable tracheal splints for survival extension in a porcine model with severe tracheobronchomalacia (TBM) [67]. The splint was fabricated with PCL and 4% hydroxyapatite using the SLS process. The interventional trachea-splinted group in the animal study showed a longer survival time than the non-splinted group. Similarly, Kaye et al. demonstrated an ex vivo tracheomalacia model for tracheal collapse using porcine tracheas and observed a significant difference between the control and tracheomalacia groups. They proved that this collapse could be treated successfully using a 3D-printed external splint [68]. More recently, Liu fabricated an external airway splint using PCL and the SLS method and tested its efficacy in dogs with tracheomalacia [22]. Before and after implantation, the stiffness of the scaffold was similar to that of their trachea. The external stent sustained a patency of 80% for 12 weeks and prolonged the life of the dogs.

To further study external splints, Ott et al. evaluated the mechanical properties of various types of scaffolds using electrospinning and 3D printing with PCL and Poly(lactic-co-glycolic acid) (PLGA) biomaterials [69]. Among them, the tri-layer scaffold with rings, which was graded as PCL and PLGA, had improved tensile and radial compression properties, largely due to the 3D-printed PCL inner rings. The electrospun PCL layer of the scaffold exhibited higher recovery properties and suture retention. Liu et al. proposed composite tracheal grafts, combining decellularized grafts with 3D-printed external splints [70]. The performance of the developed composite tracheal grafts was verified using a mouse segmental orthotopic tracheal replacement model. It was confirmed that the composite structure did not cause vascular erosion, organ damage, or inflammation, and that organ collapse did not occur and epithelialization was achieved. 

On the other hand, Chen et al. suggested a self-expandable multi-layer tracheal internal stent with anti-cancer drug [71]. They loaded paclitaxel (PTX) on the inner layer and Fe_3_O_4_ magnetic nanoparticles on the middle layer. By controlling the magnetic field, they changed temperature-responsive PTX release. Their stent showed good biosafety in rabbits and kept the airway patency for 1 month after implantation in rabbit trachea.

### 3.2. Circumferential Graft

Tubular-shaped, whole-trachea reconstruction is more difficult than the reconstruction of segmental defects because the anastomotic area is wide. Therefore, researchers have attempted to simultaneously reconstruct various constituent tissues, such as the mucous layers, blood vessels, and muscles, in addition to cartilage reconstruction. These tissues are paired and evaluated according to the interests of the researchers. First, regarding cartilage reconstruction among tracheal tissues, She et al. proposed a biomimetic PCL scaffold with a separated ring structure and a collagen sponge for long-segment tracheal replacement [72]. Their structure mimicked the native trachea both structurally and mechanically. Tracheal replacement in a rabbit model showed that the engineered biomimetic trachea demonstrated satisfactory repair. Hsieh et al. proposed a rabbit trachea-mimic scaffold using biodegradable polyurethane (PU) incorporated in a chondrogenic small molecular drug (Y27632) [73]. Their scaffold showed high restoration performance with a compression modulus similar to that of the native trachea after transplantation in nude mice for six weeks. The mesenchymal stem cells (MSCs) seeded in the scaffolds were grown into cartilage-like tissues. However, the radial thickness of the scaffold was too large compared to that of the rabbit trachea. Recently, Park et al. proposed a two-step 3D bioprinting strategy to create a trachea-mimetic construct without additional sacrificial material [21]. They used a rotational stage and curved needle to print at a high speed and wrapped a sinusoidal-patterned tubular mesh to facilitate cartilage ring reconstruction. By subcutaneous implantation in a nude mouse, they validated the cartilage regeneration capacity of their constructs.

Reconstruction of the mucosal layer is also important to discharge waste from the trachea and allow smooth breathing. Therefore, research focusing on tracheal epithelialization has also been conducted. Lee et al. compared the survival rate in rabbit segmental tracheal reconstruction models with a 3D-printed PCL scaffold between those with and without a porous membrane [74]. The rabbits with the 3D-printed scaffold reinforced with porous PCL/pluronic F127 showed longer survival than those with the scaffold without the membrane. Although the mucosal layers of the rabbit tracheas were not sufficiently regenerated, the patency was well-maintained with the 3D-printed scaffold reinforced with porous PCL/F127.

Bhora et al. utilized 3D-printed circumferential tracheal grafts with PCL external scaffolds of varying degrees (270° and 360°) and included columnar bovine dermal ECM layers with a thickness of 2 mm to evaluate the reconstruction of tracheal segments in a porcine model [75]. Two weeks after transplantation, granulation tissue was identified with partial anastomotic epithelialization, and the overall survival was between 17 and 34 days. Meanwhile, Park et al. developed a tissue-engineered tracheal graft using a combination of vat polymerization and a cell sheet for circumferential tracheal reconstruction [23]. They created a bellow-shaped graft through indirect 3D printing and attached human inferior turbinate mesenchymal stromal cell sheets to the decellularized ECM hydrogel layer on the luminal surface of the scaffold. In a rabbit model, they observed a 60% survival rate over a two-month experiment period, with epithelial tissue regenerating on the entire luminal surface. Furthermore, Park et al. also compared the mechanical properties of PCL-based tracheal frames created using four-axis FDM and a conventional three-axis deposition modeling method [76]. The trachea fabricated by the rotating-based four-axis printer exhibited higher compressive and tensile strength at a similar porosity, and the animal experiment results of the four-axis printed trachea were superior to those of the conventional scaffold.

For transplanted tissues or organs to survive and maintain their function, oxygen and nutrients must be supplied, and the connection of the vascular network is necessary for their supply. Therefore, the results of studies focusing on vascularization of tracheal grafts are also reported. Pan et al. reported that a pore diameter of 200 μm is optimum for cell adhesion, and porous PCL scaffolds with the same layer thickness had better mechanical properties than the native rabbit trachea [77]. Surface modification of porous scaffolds using nano-silicon dioxide successfully improved adhesion and proliferation. For the transplantation of scaffolds into the rabbit subcutaneous gap, PCL scaffolds initially had higher mechanical properties than those of the native trachea. Weber et al. assembled a 3D-printed tracheal frame with a porcine-derived small intestine submucosa (SIS)-ECM [78]. The SIS-ECM was wrapped inside and outside the PCL frames. In an animal study using Yorkshire pigs, a smooth transition between the native and graft trachea was observed. However, severe intraluminal granulation was also observed.

To properly function as a trachea, both the exoskeleton created by the hyaline cartilage ring and the inner respiratory mucosal epithelium must be reconstructed. Therefore, researchers have attempted to reconstruct the mucous membrane at the same time as cartilage regeneration. Gao et al. also attempted whole-segment tracheal repair using a cultured chondrocyte 3D-printed scaffold [79]. In vitro chondrocyte culture and implantation of the chondrocyte-treated scaffold in the subcutaneous tissue of nude mice revealed the generation of mature cartilage tissues. However, in whole-segment tracheal repair experiments in rabbits, all animals died within ten weeks. The most common cause of death (75%) was granulation formation in the tracheal segment and a lack of epithelial tissue formation in the tracheal lumen. Park et al. fabricated an artificial trachea containing epithelial cells and chondrocytes [18]. Using a 3D bioprinter, they accumulated five layers composed of PCL and alginate-based cell bioinks. They observed in vitro cell viability and twelve-month survival in rabbits (13/15). Regeneration of the epithelium in the tracheal lumen and the formation of cartilage islets were observed. Kim et al. combined 3D printing, electrospinning, and multicell embedding. Human bronchial epithelial cells were seeded in the inner region of the scaffold, and induced pluripotent stem-cell-derived MSCs (iPSC-MSCs) and iPSC-MSC-derived chondrocytes (iPSC-Chds) were seeded onto the outer region of the scaffold. In an in vivo study, the rabbit iPSC-Chd/iPSC-MSC co-culture group showed higher cartilage formation, thick epithelial regeneration, and cilia formation [80]. In addition, Park et al. suggested the use of an omentum as an in vivo bioreactor for culturing 3D-printed artificial tracheas [81]. Zhang et al. promoted the epithelialization of 3D-printed trachea loaded tracheal basal cells (TBCs) using mouse embryonic fibroblasts [82]. They fabricated double-layer PCL scaffold and cultured autologous chondrocytes on the outer layer of the scaffold and TBCs on the inner layer. Then, tissue-engineered trachea was pre-vascularized in vivo. Two weeks after operation, lumen surfaces of rabbits were covered by ciliated epithelia. 

Alongside the reconstruction of cartilage tissue for structural stability of the trachea, a study was conducted to secure a vascular network to ensure the long-term survival of the transplanted tissue and connectivity with the host body. Taniguchi et al. generated a scaffold-free trachea using multicellular spheroids and 3D bioprinting [19]. The trachea containing the spheroids was matured in a bioreactor and transplanted into rats. Eight weeks after transplantation, the tensile strength was twice as high as that before transplantation. The cartilage tissue and capillary-like tubes containing red blood cells were observed by histological analysis. Epithelialization was observed from day 8, but the epithelial layer did not cover the entire lumen by day 23. Small amounts of granulation tissue were observed on day 8. Gao et al. used a 3D-printed poly(L-lactic acid)(PLLA) scaffold and rabbit chondrocytes for segmental tracheal reconstruction in rabbits [83]. After fabricating the scaffold, chondrocytes were cultured in vitro, and in vivo prevascularization using muscular flaps was conducted. Upon examining transplantation using rabbits, those with prevascularized tracheas (75%) showed a higher survival rate than those with chondrocyte scaffolds (0%) with epithelialization on the luminal surface. Frejo et al. tried to reconstruct the trachea using a two-stage in vivo approach of implantation in the strap muscle and to the trachea [84]. They prepared the rabbit-chondrocyte-laden collagen/alginate bioink to regenerate cartilage, and PCL was utilized to build the supporting construct. After 9 weeks, mature cartilage was observed in the grafts in the rabbits.

In addition to the reconstruction of cartilage tissue to secure the structural stability of the trachea, a study was also conducted for the simultaneous reconstruction of cartilage and muscle tissue considering the respiratory movement of the trachea to be reconstructed. Ke et al. suggested a 3D-bioprinted tracheal construct with PCL frame cell-laden hydrogels [85]. To improve chondrogenesis and smooth muscle formation by human MSCs, hydrogel components were reconstituted. Their bioprinted constructs showed distinct improvements in the quantification of smooth muscle and cartilage formation in an in vitro study conducted for two weeks. Bioprinted constructs showed similar elastic moduli and yield stresses to those of the native trachea.

Since vascularization is essential to ensure long-term survival even after producing a trachea capable of normal function through cartilage and mucosal reconstruction, a study that considered all three aspects detailed above was also conducted. Machino et al. evaluated the potential of scaffold-free structures as tracheas using spheroids and a 3D bioprinting system [20]. The scaffold-free trachea-like tubes cultured in a bioreactor improved physical strength and tissue formation. In an in vivo study using rats, the tracheal epithelium and capillaries proliferated on day 35 after implantation. Xia et al. developed a whole-segment, tissue-engineered trachea (TET) using a 3D-printed PCL scaffold with autologous auricular cartilage cells [16]. Their TET had a higher compressive strength than the native goat trachea. The TET treatment group showed lower tissue necrosis, a long-term survival rate, and mechanical support of the airway structure. However, anastomotic stenosis could not be avoided, and one animal survived for up to 98 days. Meanwhile, Huo et al. fabricated 3D-bioprinted, trachea-integrated, cartilage-vascularized fibrous tissues using tissue-specific bioinks [86]. They prepared photo-crosslinkable cartilage-specific bioink and vascularized fibrous tissue-specific bioink, and the two inks were stacked alternately to simulate the trachea shape. At 8 weeks after the rabbit trachea transplantation, the tissue-engineered trachea showed a continuous tubular construct with a smooth lumen, and cartilage-vascularized fibrous tissue and epithelial layer were generated.

Other studies have not attempted to reconstruct a specific tissue, but various attempts related to tracheal reconstruction have been reported. To combine 3D printing and electrospinning, Kang et al. proposed a multilayer scaffold with a 3D-printed thermoplastic PU (TPU) core and electrospun polylactide outer membranes [87]. An ionic liquid-functionalized graphene oxide was incorporated into the electrospun membrane to enhance its mechanical, hydrophilic, and antibacterial effects. Their scaffolds showed effective cell infiltration, proliferation, and attachment in vitro. However, only biocompatibility was confirmed in an in vivo study using rabbits. Ahn et al. proposed a flexible 3D-printed trachea scaffold with a cell-adhesive surface [88]. They fabricated a TPU restorative trachea scaffold using flexible 3D-printed patterns. They improved the cell attachment ability by accumulating electrospun fibers using the same material. Their scaffolds showed a higher elongation ratio and rotation angle with good cell attachment. Paunović et al. synthesized biomedical inks based on poly (DLLA-co-CL) methacrylates for digital light 3D printing and fabricated bioresorbable airway internal stents [89]. They tuned the elastomeric properties of 3D-printed stents by modulating the ratio of two poly (DLLA-co-CL) methacrylates with a linear structure and a four-arm structure. A stent insertion experiment using rabbits confirmed their biocompatibility, and the stents were radiographically invisible seven weeks after insertion. Kandi et al. fabricated customized tubular structures of PCL and PU by extruding polymeric ink over a rotating predefined pattern [90]. Their tracheal structures showed superior tensile and compressive moduli compared to those of the native trachea. PCL70/PU30 showed the best biocompatibility among PCL and PU composites.

### 3.3. Segmental Graft

Encouraged by the results of the external airway splint, researchers have attempted to reconstruct damaged tracheal tissue. In particular, researchers have conducted a partial reconstruction, which is relatively less difficult than ring-shaped whole-trachea reconstruction, and focused on the mucosa, cartilage, and vascularization. For the reconstruction of the respiratory mucosa in tracheal tissue, Park et al. utilized human turbinate mesenchymal stromal cell (hTMSC) sheets to promote tracheal epithelial regeneration in a 3D-printed PCL tracheal graft [91]. The PCL tracheal graft was created using an indirect 3D printing technique that involves an inverted mold. In a non-circumferential rabbit model, mature and ciliated epithelium was formed on the luminal surface of hTMSC sheets attached to the graft four weeks after transplantation. However, the bare tracheal graft showed an immature and thin epithelium. Similarly, Jung et al. fabricated half-pipe-shaped PU tracheal scaffolds using 3D printing [92]. These tracheal scaffolds consisted of a porous inner microstructure and a nonporous outer layer. In an in vivo study using rabbits, a new epithelial lining was observed after four weeks of transplantation, and ciliated respiratory epithelium was seen after eight weeks in the lumen. The structural integrity of the scaffolds was maintained for 16 weeks.

Similar to studies of tracheal reconstruction using a circumferential graft, attempts have been made to reconstruct cartilage together with the mucous membrane. Goldstein et al. created a 3D-printed graft using poly (lactic acid) for laryngotracheal reconstruction [93]. In the in vitro study, a collagen gel with mature chondrocytes was seeded onto a graft, and the cells retained their cartilaginous properties during the three-week study period. In the in vivo study, all rabbits survived for 12 weeks. In addition, a well-mucosalized tracheal lumen and newly formed cartilage were observed. In a study by Kaye et al., a 3D-printed tracheal segment was transplanted with rabbit tracheal chondrocytes [17]. The native trachea (2 cm) was resected, and the PCL tracheal segment was transplanted. Mature cartilage and mucosal lining were generated at the end of the anastomosis; however, fibrous tissue surrounded the PCL scaffold. Bronchoscopy revealed extensive intraluminal stenosis (34.2–95%) due to intraluminal collapse and inflammatory infiltrates.

Rehmani et al. evaluated tracheal grafts using 3D-printed PCL and ECM in a porcine anterior defect model, and vascularization of the reconstructed trachea was observed [15]. In vitro tests using MSCs showed a viability of 84% with minimal cytotoxicity. In the in vivo study, five of the seven animals (71%) remained alive for the study period of 90 days. Histological analysis of the graft revealed a ciliated epithelium and vascularization of the lumen.

In addition, researchers have attempted to use novel biomaterials to reconstruct the trachea. Best et al. proposed an electrospun trachea scaffold comprising PET:PU at a ratio of 2:8 with solid C-rings of PCL for biomechanical properties and cell-seeding capacity [94]. The biomechanics of porous rings were similar to those of the native ovine trachea, and the solid C-ring scaffold showed better cell-seeding efficiency. However, additional in vitro and animal test results have not yet been reported. Maity et al. proposed shape-memory-displaying and drug-releasing tracheal stents using polypropylene glycol/PCL tri-block photocurable ink [95]. Polyethylene glycol was then covalently bonded to the stent surface to endow an antifouling effect. Ciprofloxacin was then added to the ink to confer antibacterial activity. In an in vitro study, the stent showed anti-adhesive and antibacterial properties (Table 1).

## 4. Prospect

As a result of the success of the transplantation of a 3D-printed tracheal stent into a pediatric patient in 2012, many related clinical studies for the treatment of bronchomalacia have been performed worldwide. Owing to subsequent successes, research related to the development of TET, which had been reduced due to the death of many patients after tracheal transplant surgery by Paolo Macchiarini, has been conducted again [96]. In particular, 3D printing and 3D bioprinting technologies that can produce patient-specific tracheas by utilizing patient medical images have presented new possibilities for the development of TETs. 

To use the developed TET reconstruction technology in surgery, the preparation of cell-laden bioink, and the bioprinting process should be performed in a good manufacturing practice (GMP) facility. It is also necessary to establish a stable printing process that can minimize deviations among products. In addition, the biological stability, toxicity, safety, and effectiveness of the developed tracheal construct should be evaluated and confirmed. An evaluation method using large animals equivalent to a clinical study must also be established, including long-term tracking of any side effects. 

In particular, because many cells are required for the bioprinting process to reach a clinically relevant size, it is necessary to establish stable cell sources. Human-derived stem cells or induced pluripotent stem cells, along with human epithelial cells, chondrocytes, and muscle cells, are potential cell sources. These cells should be produced, stored, and managed in a GMP facility in accordance with national guidelines.

The quality of polymeric biomaterials for 3D printing must also be maintained during the production process so that they can be used for surgery. Efforts should be made to prevent contamination during delivery. Moreover, additional sterilization procedures for entry into GMP, such as gas and radiation sterilization, should be performed. If the aforementioned problems are successfully solved, tracheal reconstruction technology using 3D printing and bioprinting could be applied to clinical research and become established as a successful patient treatment technology.

## 5. Conclusions

Here, the 3D printing and bioprinting technologies used in tracheal reconstruction have been summarized, and various research results related to the reconstruction of mucous membranes, cartilage, blood vessels, and muscle tissues, which are tissues required for tracheal reconstruction, have been classified according to the target tissue. In tracheal reconstruction using 3D printing, research advances have been summarized by dividing them into categories of structures for treatment with external splints, circumferential grafts, and segmental grafts. In addition, the issues that need to be resolved for the use of tissue-engineered grafts in clinical trials have been summarized. This review is expected to guide the development of artificial tracheas and corresponding clinical trials.

## Figures and Tables

**Table 1:** Utilization results of the auxetic structure as a tissue engineering scaffold.

Author	Fabrication Technology	Specific Fabrication Method	Material (Bio-Ink)	Target (Species)	Evaluation (Regeneration)
Zopf et al. (2013) [11]	3D Printing	SLS	PCL	External splint (human)	Tracheomalacia
Morrison et al. (2015) [12]	3D Printing	SLS	PCL/HA	External splint (human)	Tracheomalacia
Morrison et al. (2017) [13]	3D Printing	FDM	PEKK	External splint (human)	Tracheomalacia
Les et al. (2019) [14]	3D Printing	SLS	PCL/HA	External splint (human)	Tracheomalacia
Huang et al. (2016) [66]	3D Printing	FDM	PCL	External splint (human)	Tracheomalacia
Zopf et al. (2014) [67]	3D Printing	SLS	PCL/HA	External splint (porcine)	Tracheomalacia
Kaye et al. (2017) [68]	3D Printing	FDM	PLA	External splint (porcine)	Tracheomalacia
Liu et al. (2021) [22]	3D Printing	SLS	PCL	External splint (dog)	Epithelialization
Ott et al. (2016) [69]	3D Printing	FDM + Electrospinning	PCL vs. PLGA	External splint (cell)	-
Liu et al. (2022) [70]	3D Printing + decellularized graft	Vat	Biocompatible resin	External splint (mouse)	epithelialization
Chen et al. (2022) [71]	3D Printing	FDM	PCL	Internal splint (rabbit)	Tracheal stenosis
She et al. (2021) [72]	3D Printing	FDM + Coating	PCL/Collagen (Chondrocytes)	Circumferential graft (rabbit)	Cartilage formation
Hsieh et al. (2018) [73]	3D Printing	FDM	PU (hMSCs)	Circumferential graft (mouse)	Cartilage formation
Park et al. (2021) [21]	3D Bioprinting	FDM + Extrusion	PCL (hNCs, hNTSCs + Collagen)	Circumferential graft (mouse)	Cartilage formation
Lee et al. (2016) [74]	3D Printing	FDM	PCL	Circumferential graft (rabbit)	Epithelialization
Bhora et al. (2017) [75]	3D Printing	FDM	PCL	Circumferential graft (porcine)	Epithelialization
Park et al. (2018) [23]	3D Printing	Vat (indirect) + Cell sheet	PCL/Silicone	Circumferential graft (rabbit)	Epithelialization
Park et al. (2018) [76]	3D Printing	FDM	PCL	Circumferential graft (rabbit)	Epithelialization
Park et al. (2019) [18]	3D Bioprinting	FDM + Extrusion	PCL (Chondrocytes, epithelial cells + Alginate)	Circumferential graft (rabbit)	Cartilage formation, epithelialization
Machino et al. (2019) [20]	3D Bioprinting	Kenzan method	Spheroid	Circumferential graft (rat)	Cartilage formation, epithelialization, vascularization
Gao et al. (2017) [79]	3D Printing	FDM	PCL (Chondrocytes)	Circumferential graft (rabbit)	Cartilage formation, epithelialization
Kim et al. (2020) [80]	3D Printing	FDM + Electrospinning	PCL (iPSCs + Matrigel)	Circumferential graft (rabbit)	Cartilage formation, epithelialization
Park et al. (2018) [81]	3D Printing	FDM	PCL	Circumferential graft (rabbit)	Cartilage formation, epithelialization
Zhang et al. (2021) [82]	3D Printing	FDM	PCL (Chondrocytes + Matrigel)	Circumferential graft (rabbit)	Cartilage formation, epithelialization
Xia et al. (2019) [16]	3D Printing	FDM	PCL (Chondrocytes + Collagen)	Circumferential graft (goat)	Cartilage formation, epithelialization, vascularization
Taniguchi et al. (2018) [19]	3D Bioprinting	Kenzan method	Spheroid	Circumferential graft (rat)	Cartilage formation, vascularization
Gao et al. (2019) [83]	3D Printing	FDM	PLLA (Chondrocytes + Matrigel)	Circumferential graft (rabbit)	Cartilage formation, vascularization
Frejo et al. (2022) [84]	3D Printing	FDM + coating	PCL (Chondrocytes + Collagen/alginate)	Partial graft (rabbit)	Cartilage formation, vascularization
Pan et al. (2019) [77]	3D Printing	FDM	PCL	Circumferential graft (rabbit)	Vascularization
Weber et al. (2021) [78]	3D Printing	FDM	PCL/SIS-ECM	Circumferential graft (pig)	Vascularization
Ke et al. (2020) [85]	3D Printing	FDM + Extrusion	PCL (hMSCs + Collagen/ hyaluronan)	Circumferential graft (cell)	Cartilage formation, muscle formation
Huo et al. (2022) [86]	3D Bioprinting	Extrusion	(Chondrocytes, fibroblast + Decellularized hydrogels)	Circumferential graft (rabbit)	Cartilage formation, epithelialization, vascularization
Kang et al. (2019) [87]	3D Printing	FDM + Electrospinning	TPU/PLA	Circumferential graft (rabbit)	-
Ahn et al. (2019) [88]	3D Printing	FDM + Electrospinning	PCL/PU	Circumferential graft (cell)	-
Paunović et al. (2021) [89]	3D Printing	Vat	p(DLLA-co-CL)	Circumferential graft (rabbit)	-
Kandi et al. (2021) [90]	3D Printing	FDM	PCL/PU	Circumferential graft (cell)	-
Park et al. (2015) [91]	3D Printing	Vat (indirect)	PCL	Segmental graft (rabbit)	Epithelialization
Jung et al. (2016) [92]	3D Printing	FDM	PU	Segmental graft (rabbit)	Epithelialization
Goldstein et al. (2015) [93]	3D Printing	FDM	PLA (Chondrocytes + Collagen)	Segmental graft (rabbit)	Cartilage formation, epithelialization
Kaye et al. (2019) [17]	3D Bioprinting	FDM + Extrusion	PCL (Cartilage + Alginate/collagen)	Segmental graft (rabbit)	Cartilage formation, epithelialization
Rhemani et al. (2017) [15]	3D Printing	FDM	PCL/ECM	Segmental graft (porcine)	Epithelialization, vascularization
Best et al. (2017) [94]	3D Printing	FDM	PET/PU/PCL	Segmental graft (cell)	-
Maity et al. (2021) [95]	3D Printing	Vat	PCL-PPG-PCL diacrylate	Segmental graft (cell)	-

3D, three dimensional; ECM, extracellular matrix; FDM, fused deposition modeling; HA, hydroxyapatite; PCL, polycaprolactone; PEKK, polyetherketoneketone; PET, polyethylene terephthalate; PLA, poly lactic acid; PLGA, poly(lactic-co-glycolic acid); PLLA, poly(L-lactic acid); PPG, polypropylene glycol; PU, polyurethane; SIS, small intestine submucosa; SLS, selective laser sintering; TPU, thermoplastic polyurethane; hNCs, human nasal chondrocytes; hNTSCs, human nasal turbinate stem cells; hMSCs, human mesenchymal stem cells.

## Data Availability

All data used in this paper are contained within the article.
